# An Efficient Algorithm for Recognition of Human Actions

**DOI:** 10.1155/2014/875879

**Published:** 2014-08-27

**Authors:** Yaser Daanial Khan, Nabeel Sabir Khan, Shoaib Farooq, Adnan Abid, Sher Afzal Khan, Farooq Ahmad, M. Khalid Mahmood

**Affiliations:** ^1^School of Science and Technology, University of Management and Technology, Lahore 54000, Pakistan; ^2^Department of Computer Science, Abdul Wali Khan University, Mardan 23200, Pakistan; ^3^Faculty of Information Technology, University of Central Punjab, 1-Khayaban-e-Jinnah Road, Johar Town, Lahore 54000, Pakistan; ^4^Department of Mathematics, University of the Punjab, Lahore 54000, Pakistan

## Abstract

Recognition of human actions is an emerging need. Various researchers have endeavored to provide a solution to this problem. Some of the current state-of-the-art solutions are either inaccurate or computationally intensive while others require human intervention. In this paper a sufficiently accurate while computationally inexpensive solution is provided for the same problem. Image moments which are translation, rotation, and scale invariant are computed for a frame. A dynamic neural network is used to identify the patterns within the stream of image moments and hence recognize actions. Experiments show that the proposed model performs better than other competitive models.

## 1. Introduction

Human action recognition is an important field in computer vision. The implications of robust human action recognition system, requiring minimal computations, include a wide array of potential applications such as sign language recognition, keyboard or a remote control emulation, human computer interaction, surveillance, and video analysis. Such systems are developed to enable a computer to intelligently recognize a stream of complex human actions being input via a digital camera. It thrives for the need of a multitude of efficiently designed algorithms pertaining to pattern recognition and computer vision. Background noise, camera motion, and position and shape of the object are major impairment factors against the resolution to this problem. This paper presents an efficient and sufficiently accurate algorithm for human action recognition making use of image moments. A comprehensive understanding of image moments describes characteristics information of an image. The proposed system aims to recognize human actions regardless of its position, scale, colors, size, and phase of the human. The paper describes a robust feature extraction and comprehensive classification and training processes. The primary focus is to facilitate video retrieval classified on the basis of featured human action. Inherently it requires methods to identify and discover objects of interest by providing comprehensive features after video segmentation, feature extraction, and feature vector organization. These features are designed such that they are immune to encumbrances such as noise and background view. This calls for methods incessantly capable of tackling video descriptors which are repeatable and most relevant. An efficient computational paradigm for extraction of such descriptors needs to be devised because only those areas of an image are matters of concern, which contain deciphering features. A real-time implementation is realized for detection of nominated human actions. Various researchers have addressed the proposed problem using different methodologies. Tran et al. represent human action as a combination of the movements of the body part [1]. They provide a representation described by a combination of movements of the body part to which a certain action correlate. Their proposed method makes use of polar pattern of the space for representing the movement of the individual parts of the body. In another article Ali and Shah [2] represent kinematic functions computed from optical flow for the recognition of human action in video tribes. These kinematic features represent the spatiotemporal properties of the video. It further performs principal component analysis (PCA) on the kinematic feature volume. Multiple instance learning (MIL) is used for the purpose of classification of human action using succinct data after PCA. Busaryev and Doolittle recognize hand gestures captured from a webcam in real time. Such classification of gestures is applied to control real-world applications. Background subtraction and HSV-based extraction are compared as methods for getting a clean hand image for further analysis. The gesture in each hand image is then determined with Hu moments or a local feature classifier, and each gesture is mapped to a certain keystroke or mouse function [3]. Cao et al. combine multiple features for action detection. They build a novel framework which combines GMM-based representation of STIPs based detection [4]. In order to detect moving objects from complicated backgrounds, Zhang et al. improved Gaussian mixture model, which uses K-means clustering to initialize the model and gets better motion detection results for surveillance videos [5]. They demonstrate that the proposed silhouette representation, namely, “envelope shape,” solves the viewpoint problem in surveillance videos. Shao et al. present a method that extracts histogram of oriented gradients (HOG) descriptors corresponding to primitive actions prototype [6]. The output contains only the region of interest (ROI). Using this information the gradient of motion is computed for motion estimation. The gradient vectors are obtained for the partitioning of periodic effect. Once it detects a complete cycle of movement, two key frames are selected for encoding the motion. Finally, the current class action descriptors for the classification of features are extracted while the corresponding classifier is trained offline. Ullah et al. implemented the bag of features (BoF) approach for classification of human actions in realistic videos. The main idea is to segment videos into semantically meaningful regions (both spatially and temporally) and then to compute histogram of local features for each region separately [7, 8].

Certain weaknesses of the recognition algorithm for human actions in video with the kinematic features [8] and multiple instance learning are quite evident. Firstly the kinematic properties selected are not scale, translation, and rotation invariant, as the same action from different angles induces different optical flow. Secondly, occlusion presents serious consequences for the performance of the algorithm, especially in cases where a significant part of the body is closed. Moreover the training step is the slowest part of the algorithm which makes excessive use of memory due to its iterative nature. The method using the HSV model for segmentation of hands will have problems if another object of the same hue is present in the frame. Other methods using sparse representations of human action recognition cannot handle several actions in a video clip. This is because they do not take into account the spatial and temporal orientation of the extracted features. The method discussed in [9, 10] uses color intensities to segment the action by manually selecting a region. Using this approach a region must be selected every time when the scene changes; this undesirably requires human intervention. Furthermore, most of the algorithms work only for a specific illumination; it will fail to give results on high or low illumination. The approach used in [11] is based upon the assumption that each independent observation follows the same distribution. Certainly this approach is bound to fail in case the distribution of the observations is quite the reverse. Although the approach seems to be scale invariant still it is not rotation invariant.

The paper is organized into several sections.  Section 1 gives a brief introduction of the problem and the current state of the art.  Section 2 gives an overview of the proposed system.  Section 3 describes the feature extraction process.  Section 4 gives a comprehensive description of the training process.  Section 5 provides some detailed results from the model while Section 6 adds some conclusive remarks.

## 2. An Overview of the Proposed System

**Figure 1:**
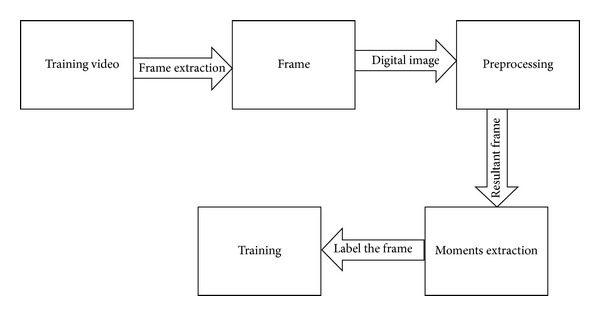
The steps of the training process.

The system is designed to retrieve semantically similar video clips for a given criterion video clip, from a large set of semantically different videos. The video dataset contains features of every proposed action and on query, video features will be extracted and matched with the stored features in the feature library. Since gestures are sequence of postures (static frames), therefore the system is expected to recognize gestures by identifying constituent postures one by one. Ultimately a temporal classifier is used to classify the input stream of spatial postures into an action.  Figure 1 shows the flow of the initial training process. Firstly, individual frames are extracted from the video input. Secondly each extracted frame is preprocessed to make it suitable for moment extraction. These moments form a feature vector which is initially used for training of the system.

**Figure 2:**
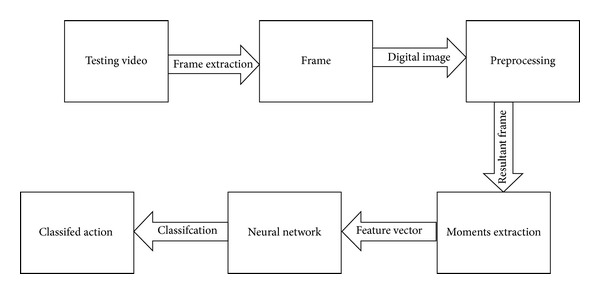
The classification process.

The system is exhaustively trained using the training process described later. A sufficiently trained system is deemed appropriate for classification of the proposed actions.  Figure 2 shows the process used for classification of human actions.

Extracted features from a live video feed are fed into a trained dynamic neural network (DNN) for the purpose of classification. The neural network classifies the action performed in a few successive frames. The dynamic neural network is designed such that its behavior varies temporally based on the video input.

## 3. Preprocessing and Feature Extraction

**Figure 3:**
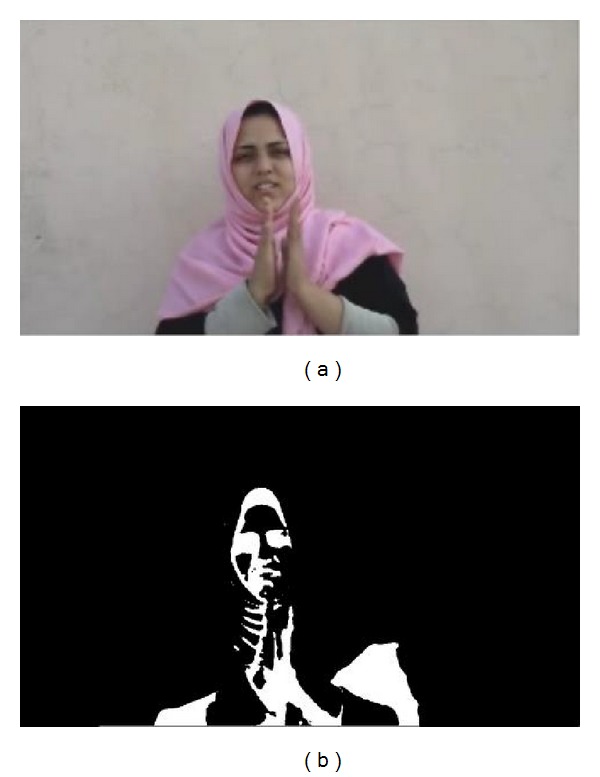
(a) The original frame. (b) The frame after.

**Figure 4:**
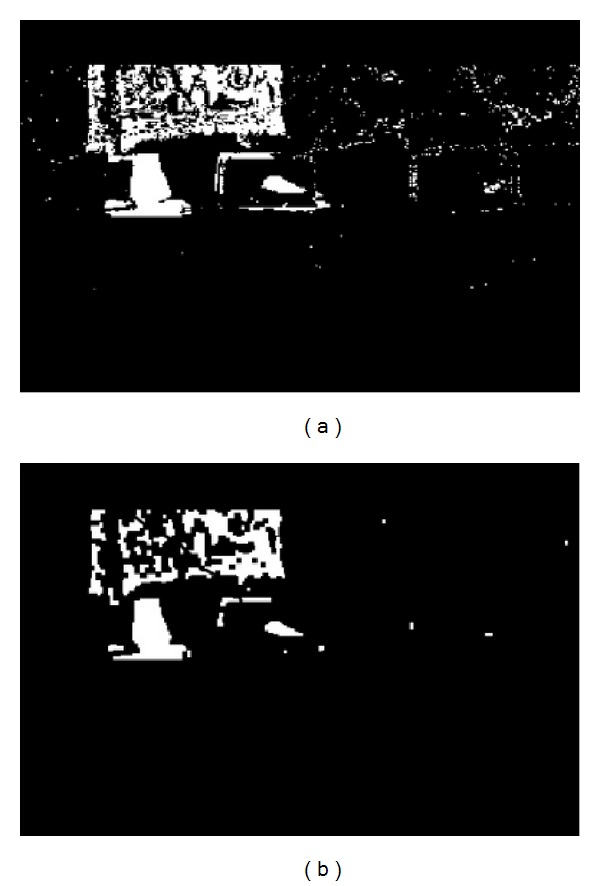
(a) The binarized image. (b) The same image after erosion dilation operations.

Initially a number of preprocessing steps must be performed on video frames before moments based features are extracted. Computations of moments require that the image is of monochrome nature. The chromatic frame extracted from the video is firstly binarized using a threshold. The threshold is carefully chosen based on the mean illumination level of the frame. Mean illumination is computed by taking the mean of luminosity value of each pixel in the frame. Once binarized, the image will hold either black or white pixels. Further to remove noise and other impairments dilation and erosion is performed [12]. Figures 3 and 4 show the result of this process on a sample frame.

Before any intricate processing is performed on the data set, the background is removed from each frame. Here two alternate approaches are adopted. In the first approach initial few frames are captured without any foreground action containing only the background. Any frame from this initial footage is used as a representative. This frame is subtracted from each frame containing foreground to obtain the filtered foreground. In the other approach each successive frame is XORed. The resultant frame represents the change in action during the period of the latter frame. The difference frame in this case also excludes the background.

### 3.1. Moments Extraction

Moments are scalar quantities which are used to categorize the shape and its features. They are computed from the shape boundary and its entire region. The concept of moments in images is quite similar to the concept of moments in physics. One major difference between the two is that image moments are inherently two-dimensional in nature. The resolution to the proposed problem is sought with the help of various moments such as raw, central, and scale invariant and rotation invariant moments along with certain corporeal properties of the image like the centroid and eccentricity. Invariant moments are those moments which are impervious to certain deformations in the shape and are most suited for comparison between two images. The scale, location, and rotation invariant moments are used to extract features regardless of size, position, and rotation, respectively.

### 3.2. Raw Moments

Raw moments are calculated along the origin of the image. Let *f*(*x*, *y*) be a function that defines an image where (*x*, *y*) are any arbitrary coordinates of the image. In case of two-dimensional continuous signal the raw moment function *M*_*pq*_ for the moment of order (*p* + *q*) is given as

(1) $$\begin{array}{c} M_{pq}=\underset{x}{\sum }\underset{y}{\sum }x^{p}y^{q}f\left(x,y\right), \end{array}$$

where *f*(*x*, *y*) is *x*th pixel along *x*-axis and *y*th pixel along *y*-axis and *p*, *q* are the *p*th and *q*th indices of the moments. These moments are computed throughout the span of the image. The raw moments provide information about properties like area and size of the image; for example, the moment *M*_00_ will give the area of object.

### 3.3. Central Moments

The moments which are invariant to translation of objects in an image are called central moments as they are computed along the centroid rather than the origin. From the equation of raw moments central moments are calculated such that the first two order moments from (18), that is, *M*_10_ and *M*_01_, are used to locate the centroid of the image.

Let *f*(*x*, *y*) be a digital image; then reducing the coordinates in previous equation by center of gravity ($\overset{-}{x}$ and $\overset{-}{y}$) of the object we get

(2) $$\begin{array}{c} \mu_{pq}=\underset{x}{\sum }\underset{y}{\sum }{(x-\overset{-}{x})}^{p}{(y-\overset{-}{y})}^{q}f\left(x,y\right). \end{array}$$

The coordinates of the center of mass $\left(\overset{-}{x},\overset{-}{y}\right)$ are the point of intersection of the lines $x=\overset{-}{x}$ and $y=\overset{-}{y}$, parallel to the *x* and *y*-axis, where the first order moments are zero. The coordinates of the center of gravity are the components of the centroid given as follows:

(3) $$\begin{array}{c} \overset{-}{x}=\frac{M_{10}}{M_{00}},\;\;\overset{-}{y}=\frac{M_{01}}{M_{00}}, \end{array}$$

while

(4) $$\begin{array}{c} {\mu_{00}=M}_{00},\\ \mu_{01}=\mu_{10}=0. \end{array}$$

Moments of order up to three are simplified in [13] and are given as follows:

(5) $$\begin{array}{c} \mu_{11}=M_{11}-\bar{x}M_{01}=M_{11}-\bar{y}M_{10},\\ \mu_{20}=M_{20}-\bar{x}M_{10},\\ \mu_{20}=M_{20}-\bar{x}M_{10},\\ \mu_{21}=M_{21}-2\bar{x}M_{11}-\bar{x}M_{20}+2\bar{x^{2}}M_{01},\\ \mu_{12}=M_{12}-2\bar{y}M_{11}-\bar{x}M_{02}+2\bar{y^{2}}M_{10},\\ \mu_{30}{=M}_{30}-3\bar{x}M_{20}-2\bar{x^{2}}M_{10},\\ \mu_{03}{=M}_{30}-3\bar{y}M_{02}-2\bar{y^{2}}M_{10}. \end{array}$$

It is shown in [14] that the generalized form of central moments is

(6) μpq=∑mp∑nq(pm)(qn)(−x¯)(p−m)(−y¯)(q−n)Mmn.

The main advantage of central moments is their invariances to translations of the object. Therefore they are suited well to describe the form or shape of the object while the centroid pertains to information about the location of the object.

### 3.4. Scale Invariant Moments

The raw moments and the central moments depend on the size of object. This creates a problem when the same object is compared but both the images are captured from different distances. To deal with this encumbrance scale invariant moments are calculated. Moments *μ*_*ij*_  are invariant to changes in scale and are obtained by dividing the central moment by scaled (00)th moment as given in the following:

(7) $$\begin{array}{c} \mu_{ij}=\frac{\mu_{ij}}{\mu_{00}^{\left(1+\left(i+j\right)/2\right)}}. \end{array}$$

### 3.5. Rotational Invariant Moments

Rotational moments are those moments which are invariant to changes in scale and also in rotation. Most frequently used are the Hu set of invariant moments:

(8) I1=η20+η02,  I2=(η20−η02)2+4η112,I3=(η30−3η12)2+(3η21−η03)2,I4=(η30+η12)2+(η21−η03)2,I5=(η30+3η12)(η30−η12) ×[(η30+η12)2−3(η21+η03)2] +(3η21−η03)(η21+η03) ×[3(η30+η12)2−(η21+η03)2],I6=(η20−η02)[(η30+η12)2−(η21+η03)2] +4η11(η30+η12)(η21+η03),I7=(η30+3η12)(η30−η12) ×[(η30+η12)2−3(η21+η03)2] −(η30−3η12)(η21+η03) ×[3(η30+η12)2−(η21+η03)2].

All the moments discussed in this section are computed for each frame. The collection of the moments is used as a feature vector. This feature vector provides characteristic information about the contents of the frame in numerical form. The variation of patterns formed by periodic frames in a video defines the action being performed. Further a framework is presented capable of recognizing the hidden patterns within the stream of feature vectors for each defined human action [14–16].

## 4. Training the Network

**Figure 5:**
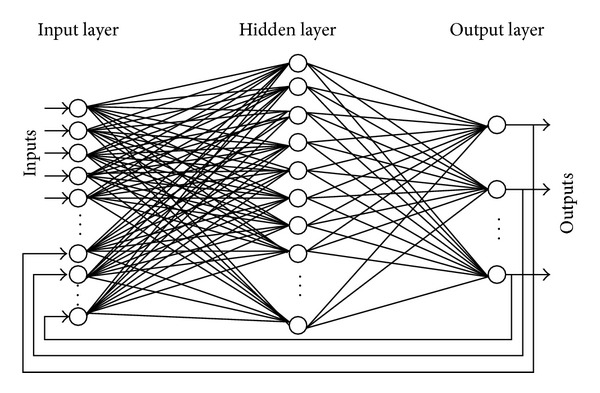
A recurrent neural network, notice the output being recurrently fed into the input layer.

A drawback of supervised training is that training data needs to be labeled. Initially each frame in the training video is assigned a class number. A specific number is assigned to each class, inherently; the frame related to any class will be given a class number. A target matrix is organized such that each column represents a label of a frame within the training data. Another input matrix is correspondingly organized in which each column contains the extracted moments of the frame. Further a neural network is designed such that neurons in the input layer could be clamped to each element in the obtained feature vector. The neurons in hidden layer are variable and will be changed to fine-tune the results, while the output layer has neurons equivalent to the number of designated classes. Moreover the network is of recurrent nature; that is, the output at output layer is recurrently clamped with the input as shown in Figure 5. Initially all the inputs and outputs of hidden layer are assigned random weights. Back propagation algorithm is used to adjust these weights and converge the output. This algorithm makes use of the sigmoid function (*σ*) for the training purpose given as

(9) $$\begin{array}{c} \sigma \left(x\right)=\frac{1}{1+e^{-x}}. \end{array}$$

The derivative of this function is given as

(10) $$\begin{array}{c} \frac{d\sigma \left(x\right)}{dx}=\sigma \left(x\right)\cdot \left(1-\sigma \left(x\right)\right). \end{array}$$

The feature vector for each frame is fed into the input layer and the output is computed. Initially randomly assigned weights are used for each edge. The difference between the actual and labeled output determines the error. Back propagation technique back-tracks this error and readjusts the weights so that the error is reduced. The weights are adjusted in a backward direction. In case of proposed network weights are adjusted in the hidden layer and then the same is done for input layer. Several iterations are performed for each input until convergence is achieved and no appreciable change in weights is perceived.

Let the weight of an edge between an arbitrary neuron *i* in input layer and an arbitrary neuron in hidden layer *j* be given as *w*_*ij*_ while the weight of an edge between an arbitrary neuron *j* in hidden and arbitrary neuron *k* in output layer is given as *w*_*jk*_. For each neuron in input layer the following operations are performed:

(11) ψj=∑i=1Nxiwij−τj,  χj=11+e−ψj,

where *N* represents the number of input layer neurons and *τ*_*j*_ the threshold used by the *j*th neuron in the hidden layer. Outputs at the hidden layer are given as follows:

(12) ψk=∑j=1Mχjwjk−τk,  χk=11+e−ψk,

while *τ*_*k*_ is the threshold of the *k*th neuron at the output layer, *χ*_*k*_ is the neuron output, and *M* is the number of neurons in hidden layer.

The obtained feature vector for a single video frame is clamped to the neural network in order to produce an output *z* ^*k*^. Here the difference between the desired and actual output is computed as the error *ϵ*_*k*_ given as

(13) $$\begin{array}{c} \epsilon_{k}=\lambda_{k}-z_{k}, \end{array}$$

while *λ*_*k*_ is the desired output.

Further error gradient is used to determine the incremental iterative change in the weight so that the actual output approaches the expected output. The error gradient is defined as the product of error and the rate of change in the actual output. Analytically it is given as

(14) $$\begin{array}{c} \delta_{k}=\epsilon_{k}\frac{\partial \chi_{k}}{\partial \psi_{k}}. \end{array}$$

Using the partial derivative of *χ*_*k*_ and putting it in above equation the following equation is formed:

(15) $$\begin{array}{c} \delta_{j}={\epsilon_{k}\chi }_{j}\cdot \left(1-\chi_{j}\right). \end{array}$$

The weight of edges between input and hidden layer also needs to be adjusted. For this purpose the error gradient for hidden layer should also be calculated. In the back propagation techniques the errors are back-tracked. The error gradient at output layer is primarily used to calculate error gradient at hidden layer. Here, the following equation is used to calculate it:

(16) $$\begin{array}{c} \delta_{j}=\chi_{j}\cdot \left(1-\chi_{j}\right)\overset{M}{\underset{k=1}{\sum }}w_{jk}\delta_{k}. \end{array}$$

Using these error gradients the renewed weights for neuron at each layer are computed. The following equations are used:

(17) $$\begin{array}{c} w_{ij}=w_{ij}+\gamma \cdot x_{i}\cdot \delta_{j},\\ w_{jk}=w_{jk}+\gamma \cdot \chi_{j}\cdot \delta_{k}, \end{array}$$

where *γ* is the learning rate. Generally it is a tiny positive value lesser than 1 and is adjustable according to the learning behavior of the network. Similarly the threshold used for computing the renewed weights should also be recalculated for the next iteration. The following equations are used to recalculate the weights:

(18) $$\begin{array}{c} \theta_{k}=\theta_{k}+\gamma \cdot \left(-1\right)\cdot \delta_{k}, \end{array}$$

(19) $$\begin{array}{c} \theta_{j}=\theta_{j}+\gamma \cdot \left(-1\right)\cdot \delta_{j}. \end{array}$$

Equations (18) and (19) represent the threshold for arbitrary neuron in output and hidden layer, respectively. This method of gradient descent is quite effective and works splendidly for almost all sorts of problems. It has the capability to minimize the error and optimize the network to provide accurate results. Although the training process is iterative, it ultimately needs to terminate. This termination condition is indicated by the convergence of results. The result is said to have converged when no appreciable change in weights is possible. This termination condition is determined using the mean square error given as

(20) $$\begin{array}{c} E=\frac{1}{M}\overset{M}{\underset{k=1}{\sum }}{{(\lambda }_{k}-z_{k})}^{2}. \end{array}$$

In the current problem a learning rate of *α* = 0.0001 was used. The output of a recurrent neural network is not only dependent on the current input but also dependent on the previous output. This recurrent nature of these networks makes them useful for problems which require a continuous input of dynamic data changing temporally as well. Identification of an action is not necessarily dependent on a single frame; rather previous and subsequent frames may also tell a story. Hence the use of recurrent network caters for the need for previous temporal information [17].

## 5. Results and Discussion

A large database of videos was collected containing hundreds of videos of varied length. Each video contained actions like

- walking,
- clapping,
- hugging,
- single hand waving,
- double hand waving,
- hand shaking.

**Figure 6:**
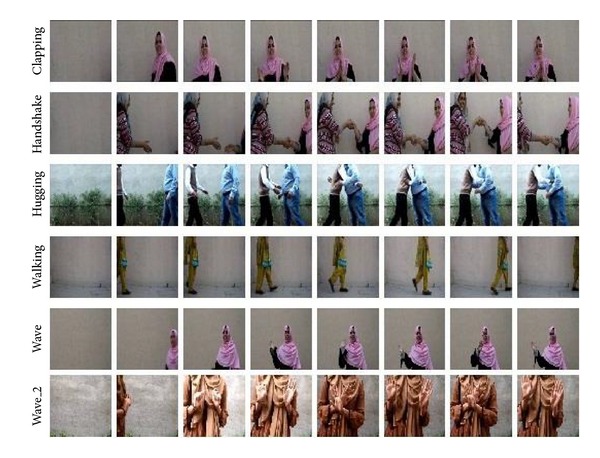
Action database: examples of sequences corresponding to different types of actions and scenarios.

**Figure 7:**
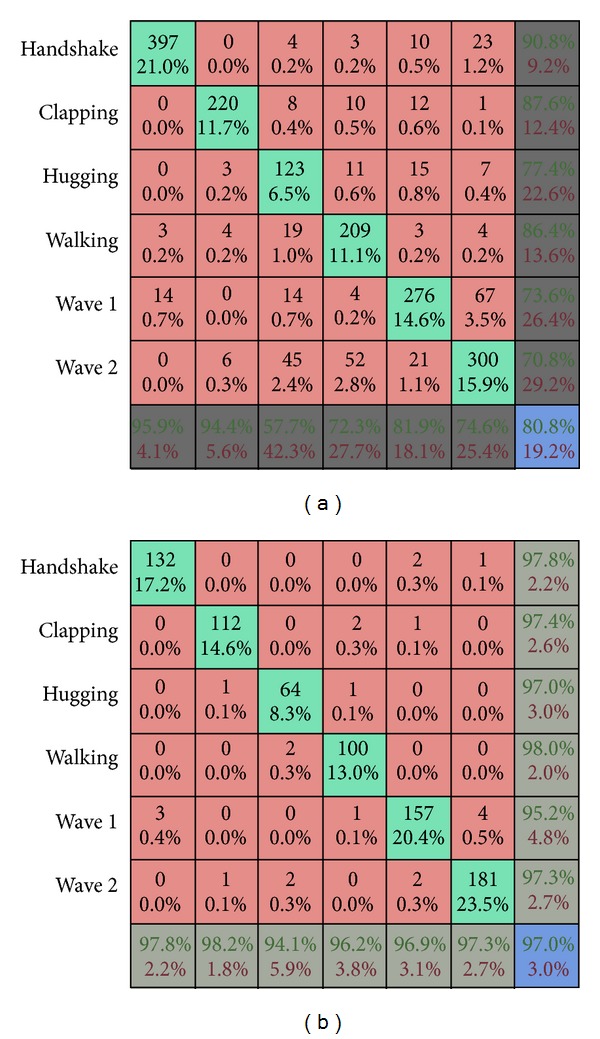
(a) The confusion matrix formed for all the frames. (b) The confusion matrix formed for medial frames.

Figure 6 shows some of the sample actions. Several videos containing different actions were taken under varied conditions in terms of illumination, location, and background. Frame by frame extraction from these videos is performed. Each frame is firstly labeled in accordance with its semantic content manually. Each stream of frames belonging to a specified class is bifurcated and kept in a folder maintaining its sequence. Hence several samples of each action are segmented from the videos manually. Each sample is a stream of videos belonging to specific action. In the next step the background or the effect of background is removed from the frame. Two different strategies are followed for this purpose. With the first method, background is removed by firstly taking a blank frame which only contains the background and then subtracting this frame from the one containing a foreground. As a result background will be eliminated. The other method used for this purpose takes the difference of two successive frames. The resultant frame will contain just the change that occurred due to motion dynamics. Once the effect of background has been countered then for all resultant frames a corresponding feature vector is formed. The feature vector of a frame contains the raw, central, scale, and rotation invariant moments of the image besides its centroid and eccentricity. Tables 1, 2, 3, 4, and 5 show the quantified values of these features. The computed vector for each frame is fed into the recurrent neural network, iteratively training the network as described previously. The training stops when the mean square error is minimized. Not all the database is used for the training purpose. One-fourth of database samples are not used for training; rather they are reserved for testing. At the point when the model has been sufficiently trained it is time to test it. The remaining samples are similarly transformed into feature vectors and fed into the trained model. The accuracy of the model is based on its ability to correctly identify these untrained samples.  Figure 7 represents the confusion matrix which shows that the overall accuracy of the system is 80.8%. Also it is noticed that the system is better able to recognize medial frames in an action rather than initial or terminal ones. The accuracy of the system is further increased to 95% if only the accuracy of recognition for the medial frames is considered.

**Figure 8:**
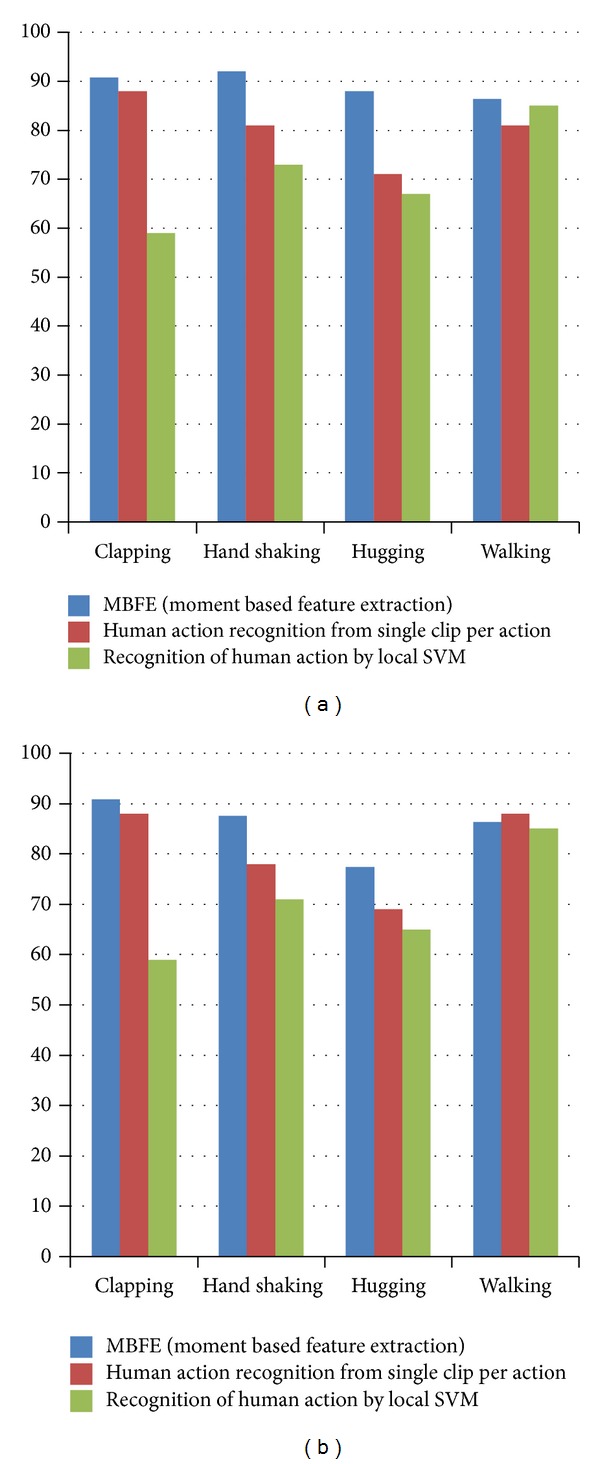
(a) The comparison of results using only the medial frames. (b) The comparison using all of the frames.

Various experiments were conducted to verify the accuracy, efficiency, and effectiveness of the system in comparison with other competitive models. A technique described in [18] extracts the features in terms of spatial as well as temporal terms. These features are used to train SVM and hence classify the video. The authors in [19] use a technique which significantly reduces the training overhead. A patch based motion descriptor and matching technique is developed by the author. A concept of transferrable learning distance is introduced which extracts the generic obscure knowledge within patches and is used to identify actions in newer videos. Both of these techniques were implemented. The accuracy of the proposed technique was evaluated in comparison with both these techniques.  Figure 8 shows the obtained results while using the assembled action database. It can be seen that the proposed technique performs reasonably well and is more consistent as compared to other competitive techniques.

**Figure 9:**
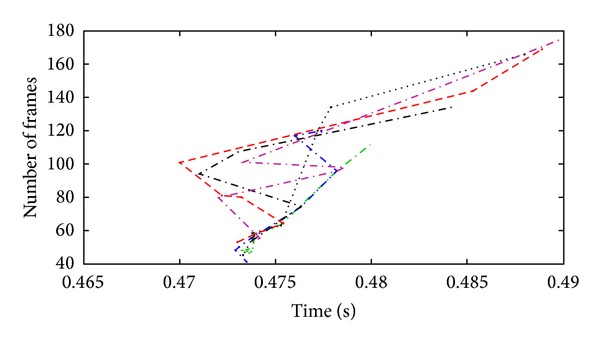
An analysis of number of frames required to identify an action.

Figure 9 somehow depicts the efficiency of the system, illustrating the number of frames against time required to classify an action. The graph shows that with the increasing number of frames the computed time for each frame length remains constant. Time required for recognition does not seem to rapidly increase if the number of frames is rapidly increased. This shows that the rapidly increasing number of frames does not have much effect on the efficiency of proposed algorithm.

**Figure 10:**
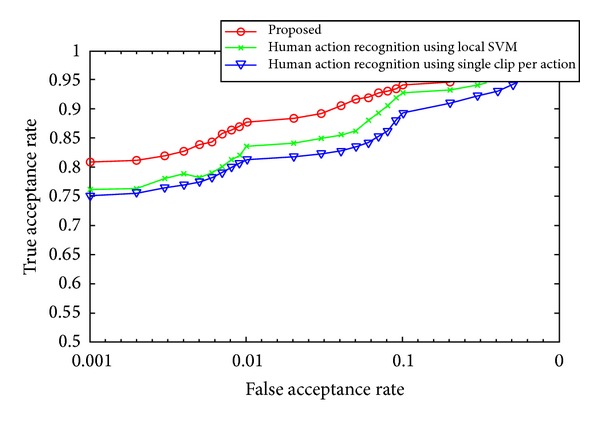
An ROC analysis for the proposed and other competitive techniques using all the frames of video.

**Figure 11:**
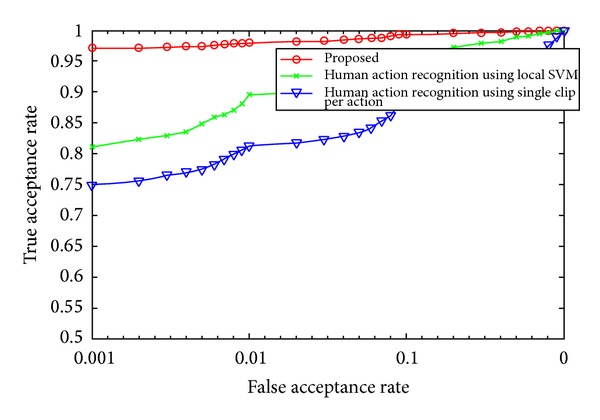
An ROC analysis for the proposed and other competitive techniques using only the medial frames of video.

A receiver operating characteristics (ROC) analysis is also performed for videos within the database.  Figure 10 gives ROC graph for all the frames in the database while Figure 11 gives the ROC distribution for only the medial frames in the video database. Both graphs suggest that the accuracy of the proposed system is better than the current state of the art. Also the accuracy is greatly increased if only the medial frames in an action video are considered.

## 6. Conclusions

The paper presents a robust technique for recognition of human actions. It provides a robust framework for feature extraction of frame and training of classifiers. The moments based feature extraction technique proves to be computationally inexpensive while providing higher accuracy than current state-of-the-art approaches. Hence it is more beneficial than other competitive techniques discussed in the paper. Experimental data exhibits that the system has an accuracy of 97% if used for medial frames. Furthermore the experimental results show that the described system is immune to acceptable illumination changes while dealing with indoor and outdoor actions.

## Figures and Tables

**Table 1:** The numerical comparison of raw moments for each of the actions.

	Clapping	Handshake	Hugging	Walking	Wave 1	Wave 2
Spatial/raw moments	1.75*E* + 03	4.90*E* + 03	7.31*E* + 03	6.70*E* + 01	1.30*E* + 03	9.47*E* + 02
	4.46*E* + 05	1.07*E* + 06	1.32*E* + 06	1.59*E* + 04	2.98*E* + 05	1.15*E* + 05
	3.91*E* + 05	8.59*E* + 05	1.55*E* + 06	6.94*E* + 03	4.10*E* + 05	3.23*E* + 05
	9.85*E* + 07	2.13*E* + 08	3.10*E* + 08	1.33*E* + 06	9.39*E* + 07	3.94*E* + 07
	1.16*E* + 08	2.63*E* + 08	2.82*E* + 08	4.16*E* + 06	7.34*E* + 07	1.63*E* + 07
	9.87*E* + 07	2.51*E* + 08	5.95*E* + 08	2.83*E* + 06	1.34*E* + 08	1.13*E* + 08
	2.53*E* + 10	5.62*E* + 10	6.85*E* + 10	3.18*E* + 08	2.32*E* + 10	5.53*E* + 09
	2.48*E* + 10	6.61*E* + 10	1.20*E* + 11	4.80*E* + 08	3.08*E* + 10	1.37*E* + 10
	3.05*E* + 10	6.86*E* + 10	6.67*E* + 10	1.14*E* + 09	1.91*E* + 10	2.70*E* + 09
	2.66*E* + 10	9.24*E* + 10	2.53*E* + 11	1.37*E* + 09	4.49*E* + 10	3.99*E* + 10

**Table 2:** The numerical comparison of central moments for each of the actions.

	Clapping	Handshake	Hugging	Walking	Wave 1	Wave 2
Central moments	1.75*E* + 03	4.90*E* + 03	7.31*E* + 03	6.70*E* + 01	1.30*E* + 03	9.47*E* + 02
	0.00*E* + 00	0.00*E* + 00	0.00*E* + 00	0.00*E* + 00	0.00*E* + 00	0.00*E* + 00
	0.00*E* + 00	0.00*E* + 00	0.00*E* + 00	0.00*E* + 00	0.00*E* + 00	0.00*E* + 00
	−8.96*E* + 05	2.57*E* + 07	3.14*E* + 07	−3.23*E* + 05	7.40*E* + 04	−1.81*E* + 04
	2.25*E* + 06	2.94*E* + 07	4.52*E* + 07	3.77*E* + 05	5.14*E* + 06	2.21*E* + 06
	1.15*E* + 07	1.01*E* + 08	2.68*E* + 08	2.11*E* + 06	4.60*E* + 06	2.28*E* + 06
	9.36*E* + 06	−1.26*E* + 09	−2.57*E* + 09	4.05*E* + 07	8.61*E* + 07	−1.40*E* + 07
	9.23*E* + 07	2.15*E* + 09	−9.86*E* + 08	−1.26*E* + 08	1.37*E* + 08	9.95*E* + 06
	−3.76*E* + 07	−1.80*E* + 09	−4.80*E* + 08	−2.51*E* + 07	−7.32*E* + 07	1.84*E* + 08
	−5.31*E* + 08	1.30*E* + 10	1.40*E* + 10	6.42*E* + 08	−1.91*E* + 08	−1.50*E* + 08

**Table 3:** The numerical comparison of image orientation for each of the actions.

	Clapping	Handshake	Hugging	Walking	Wave 1	Wave 2
Image orientation	−5.11*E* + 02	5.24*E* + 03	4.29*E* + 03	−4.82*E* + 03	5.69*E* + 01	−1.91*E* + 01
	1.29*E* + 03	6.00*E* + 03	6.18*E* + 03	5.63*E* + 03	3.95*E* + 03	2.33*E* + 03
	6.56*E* + 03	2.06*E* + 04	3.66*E* + 04	3.15*E* + 04	3.53*E* + 03	2.40*E* + 03
	−9.58*E* − 02	3.12*E* − 01	1.37*E* − 01	−1.78*E* − 01	−1.32*E* − 01	−2.49*E* − 01

**Table 4:** The numerical comparison of scale invariant moments for each of the actions.

	Clapping	Handshake	Hugging	Walking	Wave 1	Wave 2
Scale invariant	−2.91*E* − 01	1.07*E* + 00	5.87*E* − 01	−7.20*E* + 01	4.37*E* − 02	−2.02*E* − 02
	7.33*E* − 01	1.22*E* + 00	8.46*E* − 01	8.40*E* + 01	3.04*E* + 00	2.46*E* + 00
	3.74*E* + 00	4.20*E* + 00	5.01*E* + 00	4.70*E* + 02	2.72*E* + 00	2.54*E* + 00
	3.04*E* + 00	−5.27*E* + 01	−4.81*E* + 01	9.01*E* + 03	5.09*E* + 01	−1.56*E* + 01
	3.00*E* + 01	8.99*E* + 01	−1.84*E* + 01	−2.81*E* + 04	8.07*E* + 01	1.11*E* + 01
	−1.22*E* + 01	−7.50*E* + 01	−8.99*E* + 00	−5.59*E* + 03	−4.33*E* + 01	2.05*E* + 02
	−1.73*E* + 02	5.41*E* + 02	2.61*E* + 02	1.43*E* + 05	−1.13*E* + 02	−1.67*E* + 02

**Table 5:** The numerical comparison of rotation invariant moments for each of the actions.

	Clapping	Handshake	Hugging	Walking	Wave 1	Wave 2
Rotation invariants	4.47*E* + 00	5.43*E* + 00	5.86*E* + 00	5.54*E* + 02	5.75*E* + 00	5.00*E* + 00
	9.37*E* + 00	1.34*E* + 01	1.87*E* + 01	1.70*E* + 05	1.12*E* − 01	7.13*E* − 03
	4.35*E* + 04	6.08*E* + 05	1.67*E* + 05	1.97*E* + 10	1.52*E* + 05	4.41*E* + 04
	2.91*E* + 04	2.39*E* + 05	4.61*E* + 04	2.43*E* + 10	5.23*E* + 03	8.02*E* + 04
	1.02*E* + 09	8.50*E* + 10	3.89*E* + 09	5.32*E* + 20	1.17*E* + 08	−6.09*E* + 09
	8.91*E* + 04	7.40*E* + 05	1.72*E* + 05	9.97*E* + 12	−1.19*E* + 03	2.21*E* + 03
	−2.05*E* + 08	3.26*E* + 10	1.08*E* + 09	3.07*E* + 19	1.07*E* + 08	−1.96*E* + 09
	7.61*E* + 02	2.33*E* + 05	5.05*E* + 04	3.95*E* + 11	−6.43*E* + 02	3.20*E* + 03

## References

[1] Tran KN, Kakadiaris IA, Shah SK (2012). Part-based motion descriptor image for human action recognition. *Pattern Recognition*.

[2] Ali S, Shah M (2010). Human action recognition in videos using kinematic features and multiple instance learning. *IEEE Transactions on Pattern Analysis and Machine Intelligence*.

[3] Busaryev O, Doolittle J *Gesture Recognition with Applications*.

[4] Cao L, Tian YL, Liu Z, Yao B, Zhang Z, Huang TS Action detection using multiple spatial-temporal interest Point features.

[5] Zhang F, Wang Y, Zhang Z View-invariant action recognition in surveillance videos.

[6] Shao L, Ji L, Liu Y, Zhang J (2012). Human action segmentation and recognition via motion and shape analysis. *Pattern Recognition Letters*.

[7] Ullah MM, Parizi SN, Laptev I, Labrosse F, Zwiggelaar R, Liu YH, Tiddeman B Improving bag-of-features action recognition with non-local cues.

[8] deMenthon D, Doermann D Video retrieval using spatio-temporal descriptors.

[9] Volkmer T (2007). *Semantics of Video Shots for Content-Based Retrieval*.

[10] Guha T, Ward RK (2012). Learning sparse representations for human action recognition. *IEEE Transactions on Pattern Analysis and Machine Intelligence*.

[11] Jiang Z, Lin Z, Davis L (2012). Recognizing human actions by learning and matching shape-motion prototype trees. *IEEE Transactions on Pattern Analysis and Machine Intelligence*.

[12] Gonzalez RC, Woods RE (2002). *Digital Image Processing*.

[13] Flusser J, Barbara Z, Suk T (2009). *Moments and Moment Invariants in Pattern Recognition*.

[14] Flusser J, Zitova B, Suk T (2009). *Moments and Moment Invariants in Pattern Recognition*.

[15] José Antonio Martín H, Santos M, de Lope J (2010). Orthogonal variant moments features in image analysis. *Information Sciences*.

[16] Ming-Kuei H (1962). Visual pattern recognition by moment invariants. *IRE Transactions on Information Theory*.

[17] Coppin B (2004). *Artificial Intelligence Illuminated*.

[18] Schüldt C, Laptev I, Caputo B Recognizing human actions: a local SVM approach.

[19] Yang W, Wang Y, Mori G Human action recognition from a single clip per action.

